# Acceptability of Herpes Zoster Vaccination among Patients with Diabetes: A Cross-Sectional Study in Saudi Arabia

**DOI:** 10.3390/vaccines11030651

**Published:** 2023-03-14

**Authors:** Dawood Al-Orini, Abdulrahman A. Alshoshan, Abdullah O. Almutiri, Abdulsalam A. Almreef, Essa S. Alrashidi, Abdulrahman M. Almutiq, Rehana Noman, Osama Al-Wutayd

**Affiliations:** 1 Primary Healthcare Center, Al Bedaya, Ministry of Health, Qassim 56361, Saudi Arabia; 2 Research Unit, Unaizah College of Medicine and Medical Sciences, Qassim University, Unaizah 56219, Saudi Arabia; 3 Department of Family and Community Medicine, Unaizah College of Medicine and Medical Sciences, Qassim University, Unaizah 56219, Saudi Arabia

**Keywords:** acceptability, herpes zoster, vaccination, Qassim region, Saudi Arabia

## Abstract

**Background:** Vaccines have recently been made available free of charge by the Saudi Ministry of Health for people 50 years or older. Diabetes mellitus (DM) increases herpes zoster (HZ) susceptibility, severity, serious complications, and negative impacts on underlying DM conditions, which are highly prevalent in Saudi Arabia. This study aimed to assess the acceptability of the HZ vaccination and its predictors among patients with diabetes in the Qassim region of Saudi Arabia. **Methods:** A cross-sectional study of patients with diabetes from a primary healthcare center in the Qassim region was conducted. Information was obtained on sociodemographic characteristics, history of herpes zoster infection, knowing someone who had had herpes zoster, past vaccinations, and factors influencing their intention to receive the HZ vaccination through a self-administered online questionnaire. **Results:** The median age (IQR) was 56 years (53–62). Overall, 25% (n = 104/410) of the participants reported their acceptability of the HZ vaccination, and the predictors were being male (AOR 2.01, 95% CI 1.01–4.00, *p* = 0.047), believing the HZ vaccine was effective (AOR 3.94, 95% CI 2.25–6.90, *p* < 0.001), and awareness that immunocompromised individuals are at a higher risk of contracting HZ (AOR 2.32, 95% CI 1.37–3.93, *p* = 0.002). A total of 74.2% (n = 227/306) of the participants reported their acceptability of the HZ vaccination if advised by their physician, and the predictors were being male (AOR 2.37, 95% CI 1.18–4.79, *p* = 0.016) and having a history of varicella vaccine uptake (AOR 4.50, 95% CI 1.02–19.86, *p* = 0.047). **Conclusions:** One-quarter of the participants were ready to accept the HZ vaccine, but this proportion significantly increased when the patients were advised by their physicians. The uptake rate can be improved with the involvement of healthcare providers and focused awareness campaigns about the effectiveness of the vaccine.

## 1. Introduction

The Kingdom of Saudi Arabia (KSA) ranks second-highest in the Middle East for the rate of diabetes and has been reported as seventh of the top ten countries in the world by the World Health Organization (WHO), with seven million patients with diabetes and approximately three million pre-diabetic patients [1]. Patients with diabetes mellitus (DM) are highly susceptible to herpes zoster (HZ) disease [2]. The number of people living with diabetes is increasing worldwide, and according to the International Diabetes Federation (IDF), this number is estimated to reach one in ten people by 2035 [3]. In Saudi Arabia, 21.5% of HZ cases are diabetic, and diabetes is the most common comorbidity of HZ infection [4].

Herpes zoster, also called shingles, is caused by the reactivation of the varicella-zoster virus (VZV), and this is the same virus that causes varicella (chickenpox) [5].

The connection between these two infections has been acknowledged for approximately 100 years and is determined by two observations, including a primary disease with VZV, which causes varicella, and once the varicella resolves, the virus remains latent in the dorsal root ganglia and can be reactivated later in the person’s life [2,4,5].

Herpes zoster (shingles) involves the sensory ganglion, nerves, and skin [5]. It causes unilateral radicular pain and a vesicular rash, which is limited to a single dermatome, related to the sensory ganglion in which the latent VZV is reactivated [6]. Studies have indicated that approximately 25% of people develop HZ during their lifetime, and diabetes remains the main risk factor for HZ infection [7,8,9]. This is because patients with diabetes are at risk of various infections due to their impaired innate and adaptive immunity [10]. This has been affirmed by a number of studies showing that inadequate cell-mediated immunity, opsonization, and phagocytosis were weakened in patients with diabetes [11,12,13]. Another factor that plays a significant role in the reactivation of VZV among patients with diabetes is an imbalance in T-cell homeostasis [14]. Taking into account that DM is a common comorbidity with HZ and that it can lead to a substantial economic burden, the HZ vaccine can serve as a cost-effective measure [2,10,13]. Two vaccines are currently used for varicella and herpes zoster [15]. The first is the live attenuated vaccine (VZL), also known as ZOSTAVAX (OkaIMerck), which is administered in a one-dose schedule and is suitable for people aged 60 and above [16]. The other is the recombinant subunit glycoprotein E vaccine (RZV), also known as SHINGRIX, which is administered in a two-dose schedule and is suitable for people aged 50 and above [17]. SHINGRIX has been licensed by Saudi Arabia, and the Ministry of Health has recently announced its availability in all primary care centers of the Kingdom for those aged 50 and older or those aged 18 and older with immunocompromising conditions. For this reason, it is important to assess the acceptance of the herpes zoster vaccine (HZV) by patients with diabetes [18]. To the best of our knowledge, this is the first study aiming to assess the acceptability of the HZ vaccination and its predictors among patients with diabetes in the Qassim region of Saudi Arabia.

## 2. Methods

### 2.1. Study Design

A quantitative cross-sectional study was conducted at a primary healthcare center in Al Bedaya city, Qassim region of Saudi Arabia, from November to December 2022. The eligibility criteria included being 50 years or older, being registered at a primary healthcare center in Al Bedaya city, and being diagnosed with diabetes mellitus. We utilized a convenience sample, and patients who were potentially eligible received a message/call from their physician (D.A.) inviting them to participate in the study. A link was sent to those who were interested to complete a self-administered online questionnaire. The validated questionnaire was prepared according to the research objectives of this study [19,20]. It consisted of items collecting the demographic characteristics of the participants (age, gender, educational level, and employment status), along with several items about awareness of varicella, herpes zoster and its vaccine, willingness to accept the HZ vaccination, reasons for HZV hesitancy, and willingness to accept the HZ vaccination if advised by a physician. A pilot study was conducted with 25 patients with diabetes, and the results from the pilot were not included in this study. The minimum sample size was determined with OpenEpi software, and it was found to be 424. The assumptions included a 5% margin of error, a confidence interval (CI) of 95%, a level of HZ vaccination acceptance at 50% to obtain the maximum sample size, and 10% added to account for incomplete or missing responses.

### 2.2. Data Analysis

The data were analyzed using STATA software version 16. The data are presented as frequencies and percentages for the categorical variables. A simple logistic regression was performed to assess the association between the independent variables and two dependent variables (acceptability of HZ vaccination and acceptability of HZ vaccination if advised by a physician), and variables with a *p*-value of <0.25 were included in the multiple logistic regression. The crude and adjusted odds ratios in the simple and multiple logistic regression analysis models are reported, respectively. A two-sided *p*-value of ≤0.05 was considered strong evidence against the null hypothesis.

## 3. Results

**Figure 1:**
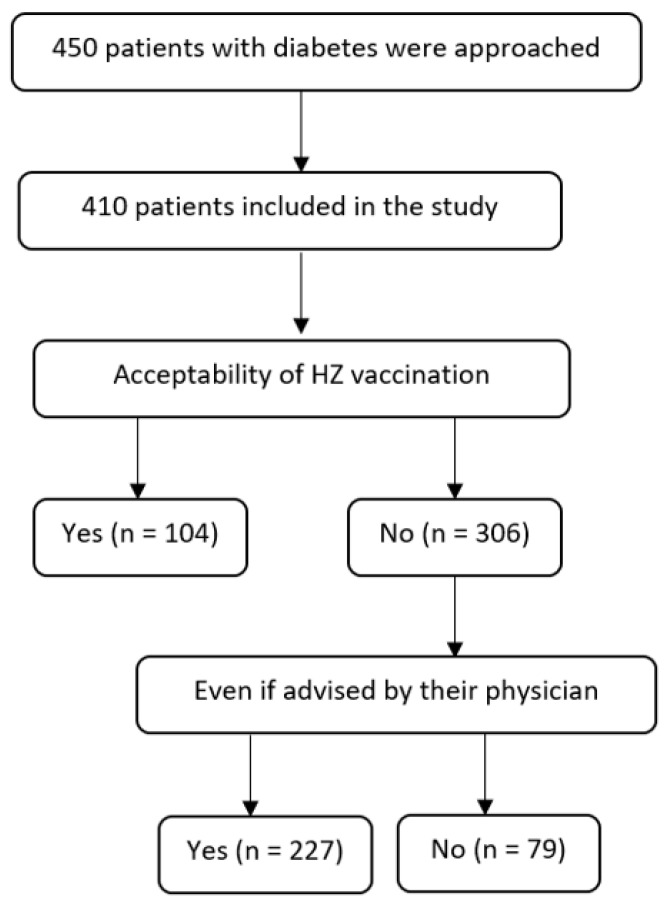
Flow diagram for patients with diabetes, Qassim, Saudi Arabia.

The response rate was 91.7%. We approached 450 participants and obtained 410 complete responses (Figure 1).

### 3.1. Acceptability of HZ Vaccine

#### 3.1.1. Participant Characteristics

A total of 410 participants were included in this study; the median (IQR) age of the participants was 56 (53–62) years, 270 (66%) were male, 260 (63.4%) had university-level or higher education, and 115 (28%) were employed. A majority of the participants (n = 351, 85.6%) knew that there was a disease called varicella, approximately one-third (n = 151, 36.8%) had had varicella disease in the past, and only a few had received the varicella vaccine (n = 35, 8.5%). Just over half of the participants knew about herpes zoster (shingles) infection (n = 234, 57%), only 23 (6%) had a previous history of HZ, and 251 (61%) knew someone who had been infected with HZ. Half of the participants (n = 219, 53%) were acquainted with the availability of the HZ vaccine, but only 82 (20%) believed that it was effective. Only 27 (6.9%) knew that an individual was at high risk of contracting HZ if they had suffered from chickenpox in the past, and approximately one-third (n = 138, 33.7%) were aware that immunocompromised individuals were at a higher risk of contracting HZ than immunocompetent individuals (Table 1).

#### 3.1.2. Predictors of Vaccination Acceptability

Overall, 25% (n = 104/410) of the participants were willing to get the HZ vaccine. According to the bivariate analysis, participants were more likely to be willing to get the HZ vaccine if they were male (COR 1.67, 95% CI 1.02–2.74, *p* = 0.043), knew about HZ infection (COR 1.68, 95% CI 1.06–2.68, *p* = 0.028), knew someone who had been infected with HZ (COR 1.69, 95% CI 1.05–2.73, *p* = 0.031), knew that there was an HZ vaccine (COR 1.94, 95% CI 1.22–3.08, *p* = 0.005), believed that the HZ vaccine was effective (COR 5.17, 95% CI 3.09–8.67, *p* < 0.001), knew that people were at higher risk of contracting HZ if they had had chickenpox (COR 4.13, 95% CI 1.86–9.15, *p* < 0.001), and knew that immunocompromised individuals were at a higher risk of contracting HZ (COR 3.19, 95% CI 2.01–5.05, *p* < 0.001).

In the multivariable analysis, the variables that remained statistically significant predictors were being male (AOR 2.01, 95% CI 1.01–4.00, *p* = 0.047), having a belief that the HZ vaccine was effective (AOR 3.94, 95% CI 2.25–6.90, *p* < 0.001), and knowing that immunocompromised individuals are at a higher risk of contracting HZ (AOR 2.32, 95% CI 1.37–3.93, *p* = 0.002) (Table 2).

#### 3.1.3. Reasons for HZ Vaccination Hesitancy

The reasons for vaccine hesitancy listed by our study respondents included concerns about side effects (31.6%), self-perceived immunity from HZ (25.4%), generally not being in favor of vaccination (14.4%), doubts about HZ vaccine effectivity (9.5%), a belief that HZ is not a serious and severe infection (6.9%), low prioritization of the HZ vaccination (3.3%), and others (8.9%).

### 3.2. Acceptance of HZ Vaccine if Advised by a Physician

#### 3.2.1. Participant Characteristics

A total of 306 participants were asked about their acceptability of the HZ vaccine if advised by their physician. Their median (IQR) age was 56 (53–61) years, 193 (63%) were male, 190 (62%) had university-level or higher education, and 85 (27.8%) were employed. The majority of the participants (n = 260, 85%) knew that there was a disease called varicella, approximately half (n = 107, 35%) had had varicella disease in the past, and only a few had received the varicella vaccine (n = 26, 8.5%). Just over half of the participants knew about herpes zoster (shingles) infection (n = 165, 53.9%), very few (n = 14, 4.6%) had a history of HZ, and most (n = 178, 58%) knew someone who had been infected with it. Half of the participants (n = 151, 49.3%) knew about the availability of the HZ vaccine, but not many (n = 38, 12.4%) believed that it was effective. Only a few (n = 12, 3.9%) knew that an individual was at high risk of contracting HZ if they had suffered from chickenpox in the past, and approximately one-quarter were aware that immunocompromised individuals were at a higher risk of contracting HZ than immunocompetent individuals (n = 82, 26.8%) (Table 1).

#### 3.2.2. Predictors of Vaccination Acceptability

A total of 71.5% (n = 445/622) of the participants were willing to get the HZ vaccine if advised by their physician. The bivariate analysis revealed that being male (COR 2.16, 95% CI 1.28–3.65, *p* = 0.004), having a history of varicella vaccine (COR 4.55, 95% CI 1.05–19.72, *p* = 0.043), knowing someone infected with HZ (COR 0.56, 95% CI 0.32–0.96, *p* = 0.034), having had an HZ infection (COR 0.24, 95% CI 0.10–0.72, *p* = 0.011), and knowing about the HZ vaccine (COR 0.58, 95% CI 0.34–0.97, *p* = 0.037) were significantly associated with vaccine acceptance. In the multivariable analysis, the variables that remained statistically significant predictors were being male (AOR 2.37, 95% CI 1.18–4.79, *p* = 0.016) and a history of varicella vaccine uptake (AOR 4.50, 95% CI 1.02–19.86, *p* = 0.047) (Table 3).

## 4. Discussion

To the best of our knowledge, this is the first study conducted in the Kingdom of Saudi Arabia to investigate the predictors of accepting a herpes zoster vaccination among patients with diabetes using an objective instrument. Our results demonstrate that one-quarter of the participants had the intention to receive the HZ vaccine in the Qassim region of Saudi Arabia. Although this rate is low, it is still higher than the rate of 4.5% in Riyadh and 18% in the whole Kingdom among patients with a history of HZ [4,21]. Our findings are consistent with the coverage rate in Greece, which was reported as 26.3% for the HZ vaccine among individuals with diabetes aged 60 years and older [22]. A wide range of variations have been noted in HZ vaccination coverage in different countries. A coverage rate of 24% was found in the USA among adults aged 60 years or older [23], and coverage rates of 9%, 11.9%, and 16.57% have been reported in South Korea, Texas, and China, respectively [24,25,26]. The uptake rate in the Netherlands has been reported as 58.1% [27].

One explanation for the low willingness to accept the HZ vaccine among our respondents, despite its free availability at PHCs in Saudi Arabia, could be the patients’ low awareness about HZ infection, the HZ vaccine, indications for patients with diabetes, and the vaccine’s free-of-cost availability. Approximately half of our participants knew about HZ, and an equal proportion was acquainted with its vaccine (HZV), while less than one-quarter thought that the vaccine was effective. These results are similar to a study conducted in the United Arab Emirates, which showed 60% awareness of HZ and 15% awareness of the vaccine [28]. Similarly, in Hong Kong, 47.1% of the study’s respondents were aware of HZV [19]. In a South Korean study and systemic review of 17 countries, 85.7% and 67.1% of the participants were aware of HZ, respectively, and 43.6% of the participants knew about the HZ vaccine [29,30]. Most people appear to have relatively limited knowledge about the HZ vaccine’s free-of-cost availability at all PHCs in the Kingdom, which was announced relatively recently in Saudi Arabia [18]. Additionally, unawareness of the indications of the specific vaccine among the older population with T2D is common, despite the fact that two-thirds of HZ cases are found at ages >50 years [31]. Our data revealed that low awareness was not the sole reason for low willingness to accept the HZV among patients with diabetes, but it was certainly a prevalent reason, as was evident in the participant responses that indicated feeling that HZV was not needed, self-perceived immunity from HZ, beliefs that HZ was not a serious or severe infection, and low prioritization of the HZ vaccination. These findings are in line with the results of the US 2007 National Immunization Survey-Adult (NIS-Adult), which reported similar findings [32]. Our data also indicated that doubts about HZV effectivity and concerns about side effects contributed a great deal to unwillingness toward the vaccination, despite the strong evidence provided by a number of studies about the safety, effectiveness, and good tolerability profile of HZV among both the <60 and >60 age groups [33,34,35]. This study also found that a certain proportion of participants were generally not in favor of vaccination, which was consistent with an Italian study conducted with the same objectives in 2016 [20]. One possible explanation for this could be people’s mistrust of vaccinations, which could lead to concerns about their possible side effects and effectiveness. We found that about 74% of those who did not intend to vaccinate against HZ were ready to receive it if advised to do so by their physicians. In other words, the rate of willingness to vaccinate against HZ was found to improve dramatically from one-quarter to half of the study participants if doing so was recommended by their physicians. This finding is consistent with previous studies [28,29]. Our data supported findings from other studies and added insights by highlighting a number of significant factors that had a positive association with HZ vaccination willingness, including male gender, an awareness that immunocompromised individuals are at a higher risk of contracting HZ, and the belief that the vaccine was effective. Moreover, we found that willingness to receive the HZ vaccine on a physician’s advice was significantly associated with the male gender and a history of varicella vaccine uptake. Our findings regarding the positive influence of physicians’ recommendations on willingness to receive the HZV are similar to studies conducted in Italy, the United Arab Emirates, South Korea, the United Kingdom, and the USA. This information is reassuring because it is plausible that physicians can play an effective role in increasing HZ vaccine coverage [20,24,28,29,36,37,38].

In contrast to a study conducted in China, we found that male participants were more likely to accept the HZV than female participants [26]. Our study provides information regarding the strong association of HZV willingness among patients with diabetes who are aware that immunocompromised individuals are at a higher risk of HZ infection and believe that the vaccine is effective. These findings correspond to those of previous studies [20,27]. This could be rationalized by our participants being more motivated toward prevention and immunization which is driven by self-perceived susceptibility and effectiveness of the vaccine as stated by the health belief model that people become more receptive to optional vaccines if they believe the following: a health condition is serious, they are susceptible to it, the vaccine would benefit them by mitigating their risk of contracting a disease, or the benefits of the vaccine are greater than its potential risks [39,40]. Despite the fact that all people aged ≥50 years should receive the HZ vaccine, regardless of their previous vaccination for varicella, the patients with diabetes who had received the varicella vaccine in the past were hesitant to receive the HZ vaccine, and they were willing to receive it only with their physician’s advice [41]. This finding can be attributed to the misconception of our participants that they were immune to shingles if they had had the varicella vaccine in the past. However, it was positive that they were largely willing to receive the HZV if their physicians recommended it to them.

The study had some limitations, including the self-reported nature of our data. Even though the accuracy of most of the responses can be ensured, it is difficult to verify participants’ responses regarding vaccine uptake or knowing someone with HZ. Additionally, we cannot rule out recall bias. A causal relationship cannot be established due to the cross-sectional study design. Further, this study has not covered some variables such as diabetes drugs, metabolic control, history of obesity, micro- or macro-vascular complications, and sleep disturbances leading to immune dysfunction, which may influence the decisions of patients with diabetes towards vaccination [42,43].

## 5. Conclusions

Willingness to receive the HZ vaccination among patients with diabetes is far below the optimum level in the Qassim region of Saudi Arabia, but it can be significantly increased if patients are advised by their physicians. We believe that our findings related to low willingness can contribute to the improvement in the HZ vaccination rate among people with DM in the KSA, but there is a need for a large-scale study to generalize the findings to all regions of the Kingdom. This will help policy-makers monitor and plan adequately at a national level. Our results support the idea that the HZV uptake rate can be improved with the involvement of healthcare providers and focused awareness campaigns about the effectiveness of the vaccine.

## Figures and Tables

**Table 1:** Descriptive characteristics of the participants.

	Acceptability of HZ Vaccination		Acceptability of HZ Vaccination if Advised by Their Physician	
	Total (n = 410)	Yes n (%) 104 (25.4)	Total (n = 306)	Yes n (%) 227 (74.2)
Age (years)				
Median [IQR]	56 (53–62)		56 (53–61)	
Sex				
Male	270	77 (28.5)	193	154 (79.8)
Female	140	27 (19.3)	113	73 (64.6)
Education				
<university	150	34 (22.7)	116	86 (74.1)
≥university	260	70 (26.9)	190	141 (74.2)
Occupation				
Unemployed	61	11 (18)	50	33 (66)
Employed	115	30 (26.1)	85	67 (78.8)
Retired	234	63 (26.9)	171	127 (74.3)
Do you know the disease called varicella?				
Yes	351	91 (25.9)	260	194 (74.6)
No	59	13 (22)	46	33 (71.7)
Have you had varicella in the past?				
Yes	151	44 (29.1)	107	83 (77.6)
No	133	31 (23.3)	102	77 (75.5)
Do not remember	126	29 (23)	67	67 (69.1)
Have you been vaccinated against varicella?				
Yes	35	9 (25.7)	26	24 (92.3)
No	180	47 (26.1)	133	97 (72.9)
Do not remember	195	48 (24.6)	147	106 (72.1)
Do you know the disease called shingles?				
Yes	234	69 (29.5)	165	117 (70.9)
No	176	35 (19.9)	141	110 (78)
Do you know someone who has had shingles?				
Yes	251	73 (29.1)	178	124 (69.7)
No	159	31 (19.5)	128	103 (80.5)
Have you had shingles in the past?				
Yes	23	9 (39.1)	14	6 (42.9)
No	387	95 (24.6)	292	221 (75.7)
There is a vaccine for shingles.				
Yes	219	68 (31.1)	151	104 (68.9)
No	191	36 (18.9)	155	123 (79.4)
Do you think that vaccines are effective for prevention?				
Yes	82	44 (53.7)	38	30 (79)
No	328	60 (18.3)	268	197 (73.5)
If an individual has chickenpox, he/she will be at risk of contracting HZ.				
Yes	27	15 (55.6)	12	8 (66.7)
No	383	89 (23.2)	294	219 (74.5)
Immunocompromised individuals are at a higher risk of contracting HZ.				
Yes	138	56 (40.6)	82	59 (72)
No	272	48 (17.7)	224	168 (75)

**Table 2:** Bivariate and multivariable analyses exploring the predictors of acceptability of HZ vaccination among diabetic patients.

Variables	Bivariate Analysis	*p*-Value	Multivariable Analysis	*p*-Value
	COR (95% CI)		AOR (95% CI)	
Age	1.01 (0.98–1.05)	0.541		
Sex				
Female	Reference		Reference	
Male	1.67 (1.02–2.74)	0.043	2.01 (1.01–4.00)	0.047
Education				
<university	Reference			
≥university	1.26 (0.79–2.01)	0.341		
Occupation				
Employed	Reference		Reference	
Unemployed	0.62 (0.29–1.35)	0.231	0.84 (0.30–2.38)	0.741
Retired	1.04 (0.63–1.73)	0.868	1.01 (0.57–1.81)	0.967
Knowing the varicella disease				
No	Reference			
Yes	1.24 (0.64–2.40)	0.526		
History of varicella infection				
No	Reference		Reference	
Yes	1.36 (0.87–2.15)	0.181	1.27 (0.76–2.12)	0.360
History of varicella vaccine				
No	Reference			
Yes	1.02 (0.46–2.25)	0.960		
Knowing about shingles				
No	Reference		Reference	
Yes	1.68 (1.06–2.68)	0.028	1.23 (0.65–2.33)	0.526
Knowing someone who had shingles				
No	Reference		Reference	
Yes	1.69 (1.05–2.73)	0.031	1.19 (0.62–2.27)	0.597
History of shingles				
No	Reference		Reference	
Yes	1.97 (0.83–4.71)	0.124	1.54 (0.54–4.39)	0.418
Knowing that there are HZ vaccines				
No	Reference		Reference	
Yes	1.94 (1.22–3.08)	0.005	1.27 (0.75–2.14)	0.378
HZ vaccines are effective				
No	Reference		Reference	
Yes	5.17 (3.09–8.67)	<0.001	3.94 (2.25–6.90)	<0.001
At risk of contracting HZ if you had chickenpox				
No	Reference		Reference	
Yes	4.13 (1.86–9.15)	<0.001	1.31 (0.50–3.42)	0.581
Immunocompromised individuals are at a higher risk of contracting HZ				
No	Reference		Reference	
Yes	3.19 (2.01–5.05)	<0.001	2.32 (1.37–3.93)	0.002

**Table 3:** Bivariate and multivariable analyses exploring the predictors of acceptability of HZ vaccination if advised by their physician among diabetic patients.

Variables	Bivariate Analysis	*p*-Value	Multivariable Analysis	*p*-Value
	COR (95% CI)		AOR (95% CI)	
Age	0.98 (0.94–1.01)	0.219	0.97 (0.93–1.01)	0.114
Sex				
Female	Reference		Reference	
Male	2.16 (1.28–3.65)	0.004	2.37 (1.18–4.79)	0.016
Education				
<university	Reference			
≥university	1.00 (0.59–1.70)	0.989		
Occupation				
Employed	Reference		Reference	
Unemployed	0.52 (0.24–1.14)	0.103	1.55 (0.55–4.39)	0.406
Retired	0.78 (0.42–1.45)	0.424	1.20 (0.59–2.45)	0.612
Knowing the varicella disease				
No	Reference			
Yes	1.16 (0.58–2.33)	0.681		
History of varicella infection				
No	Reference			
Yes	1.32 (0.76–2.29)	0.322		
History of varicella vaccine				
No	Reference		Reference	
Yes	4.55 (1.05–19.72)	0.043	4.50 (1.02–19.86)	0.047
Knowing about shingles				
No	Reference		Reference	
Yes	0.69 (0.41–1.16)	0.158	1.20 (0.61–2.38)	0.599
Knowing someone who had HZ				
No	Reference		Reference	
Yes	0.56 (0.32–0.96)	0.034	0.68 (0.35–1.34)	0.266
History of shingles				
No	Reference		Reference	
Yes	0.24 (0.10–0.72)	0.011	0.33 (0.09–0.60)	0.065
Knowing that there are HZ vaccines				
No	Reference		Reference	
Yes	0.58 (0.34–0.97)	0.037	0.63 (0.36–1.09)	0.100
HZ vaccines are effective				
No	Reference			
Yes	1.35 (0.59–3.09)	0.475		
At risk of contracting HZ if you had chickenpox				
No	Reference			
Yes	0.68 (0.20–2.34)	0.546		
Immunocompromised individuals are at a higher risk of contracting HZ				
No	Reference			
Yes	0.86 (0.48–1.51)	0.590		

## Data Availability

The data presented in this study are available on request from the corresponding author.
